# Metastatic Type II Papillary Renal Cell Carcinoma With Recurrent Complete Responses to Sunitinib: A Case Report With a Literature Review

**DOI:** 10.7759/cureus.25541

**Published:** 2022-05-31

**Authors:** Bayan H Al Ashour, Faisal Azam, Fahad Ibnshamsah, Fahad Alrowais, Ayed Al-Garni, Humaid O Al-Shamsi, Nedal Bukhari

**Affiliations:** 1 Collage of Medicine, Imam Abdulrahman Bin Faisal University, Dammam, SAU; 2 Department of Medical Oncology, King Fahad Specialist Hospital, Dammam, SAU; 3 Department of Internal Medicine, Imam Abdulrahman Bin Faisal University, Dammam, SAU; 4 Department of Radiation Oncology, King Fahad Specialist Hospital, Dammam, SAU; 5 Department of Pathology and Laboratory Medicine, King Fahad Specialist Hospital, Dammam, SAU; 6 Depatment of Oncology, Emirates Oncology Society, Dubai, ARE; 7 Department of Oncology, Burjeel Cancer Institute, Abu-Dhabi, ARE; 8 College of Medicine, University of Sharjah, Sharjah, ARE

**Keywords:** complete response, sunitinib, tyrosine kinase inhibitors, papillary, renal cell carcinoma

## Abstract

Papillary renal cell carcinoma (PRCC) is a less common subtype of kidney cancer and is typically more resistant to systemic treatments. This report describes a patient with metastatic type II PRCC who experienced two complete responses (CR) to the tyrosine kinase inhibitor (TKI) sunitinib. The patient remains on sunitinib with durable control of the disease. To the best of our knowledge, this is the first case of metastatic type II PRCC with CR to sunitinib.

## Introduction

Clear cell renal cell carcinoma (RCC) is the most common type of RCC; it represents 75% to 80% of all RCC cases. Papillary RCC (PRCC) represents a distinguished class of non-clear cell RCC and is a less common variant than clear cell RCC, accounting for 15% of cases, and is commonly resistant to conventional treatments like immunotherapy and targeted treatments [1]. PRCC is classified into two types, and type II is typically associated with resistance to systemic treatments and poorer prognosis than type I PRCC and clear cell RCC [2].

Surgery is the treatment of choice for localized and locally advanced PRCC, while advanced or metastatic PRCC is often managed with systemic treatments. Treatment classes include tyrosine kinase inhibitors (TKIs) like antivascular endothelial growth factors (anti-VEGF) and immune checkpoint inhibitors (ICI) as initial therapy. Sunitinib, an anti-VEGF that inhibits several tyrosine kinase receptors involved in cancer growth, metastasis, and neoangiogenesis, is an effective treatment for patients with clear cell RCC. PRCCs are typically more resistant to TKIs and immunotherapy [3-5]. We present a case of a patient with metastatic type II PRCC on intermittent sunitinib for almost three years with two complete responses (CRs) [1-3].

## Case presentation

**Figure 1:**
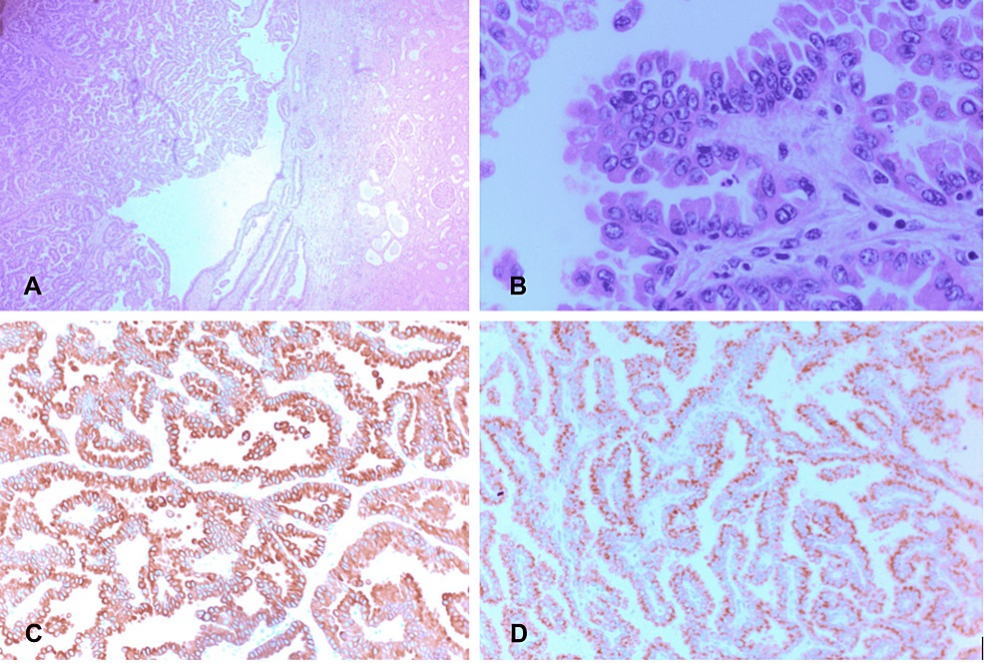
Type II papillary renal neoplasm composed of arborizing papillae lined by atypical epithelial cells with moderate eosinophilic cytoplasm and large vesicular nuclei containing prominent nucleoli. Tumor cells show diffusely positive immunostaining for cytokeratin 7 and α-methylacyl-CoA-racemase. (A) Papillary renal cell carcinoma showing arborizing papillae with adjacent non-neoplastic renal tissue. (B) Higher power image showing that papillae are lined by large cells with abundant eosinophilic cytoplasm, atypical nuclei with prominent nucleoli (ISUP Grade 3). Well-controlled immunostains show that tumor cells are variably positive for Cytokeratin 7 (C) and α-methylacyl-CoA-racemase (D). ISUP: International Society of Urologic Pathologists.

A 69-year-old man was referred to our clinic from another health care facility. He has a past medical history of longstanding hypertension and type 2 diabetes. One year prior to presentation, he underwent an open partial left nephrectomy for a 4.5-cm mass. The pathology of the resected mass was consistent with type II PRCC. The patient recovered and was regularly monitored by his urologist (Figure 1). Several months after the nephrectomy, he developed end-stage renal disease and began hemodialysis three times per week.

**Figure 2:**
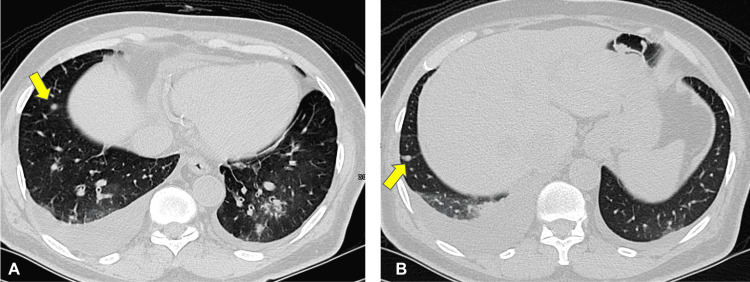
Baseline computed tomography images (A, B) just before starting treatment in late January 2019 shows multiple bilateral solid pulmonary nodules, largest measures 0.7 cm, highly suspicious for metastatic disease with bilateral pleural effusion.

A year after his surgery, he underwent a computed tomography (CT) scan that revealed metastatic retroperitoneal lymph nodes and bilateral pulmonary metastasis (Figure 2). The patient was evaluated by the medical oncology team and started on sunitinib 25 mg two weeks on and one week off. The dose was gradually increased to 37.5 mg two weeks on, followed by one week off treatment (i.e., a 21-day cycle). He experienced palmar-plantar erythrodysesthesia (grade 2) and headaches on this dosing regimen.

**Figure 3:**
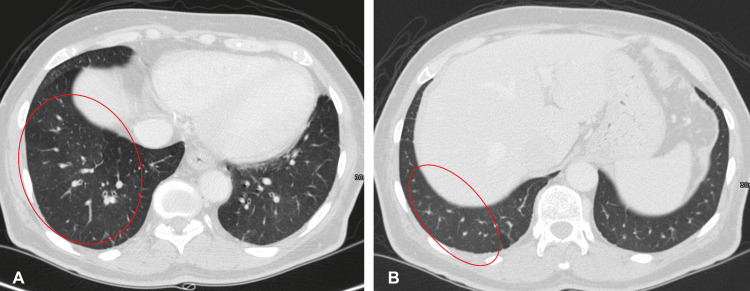
Follow-up computed tomography images (A, B) six months after starting sunitinib; shows complete resolution of lung nodules.

A CT scan six months after the start of the sunitinib regimen showed a CR of his metastatic pulmonary nodules and retroperitoneal lymph nodes. He continued sunitinib for several more months, then elected to pause treatment based on the imaging results and personal preference. He was monitored via follow-up with CT scans every four months (Figure 3).

**Figure 4:**
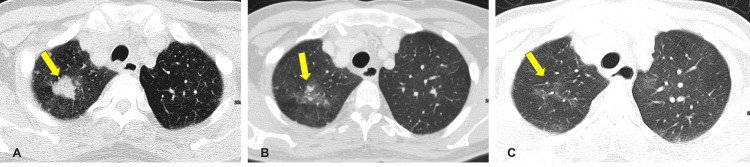
(A) Computed tomography from August 2020 (approximately eight months after discounting sunitinib): the reappearance of bilateral pulmonary nodules, largest at the right upper lobe measuring 3 cm. (B) Computed tomography repeated three months later showed a significant regression of the right upper lobe metastatic lesion. (C) Most recent computed tomography revealing complete resolution of the metastatic pulmonary nodules.

During follow-up, we noted recurrent disease involving his right upper lobe of the lung. We reintroduced sunitinib at 37.5 mg. An interval regression of this disease was noted on his following CT scan (Figure 4A-4B). He continued to experience good clinical and radiological responses. His most recent evaluation via positron emission tomography, two years since discovering the disease in his right upper lobe of the lung, confirmed his disease was in its second CR. The patient continues his 37.5 mg daily dose of sunitinib for two weeks on and one week off (Figure 4C). His future follow-up plan will be to repeat the imaging every four months and act accordingly.

## Discussion

RCC is the sixth and ninth most diagnosed cancer in men and women, respectively [4,5]. It is one of the more lethal urological malignancies originating from the renal cortex. Risk factors include smoking, alcohol consumption, obesity, comorbidities such as hypertension and chronic kidney disease, prolonged use of drugs such as antihypertensive medication, and environmental causes. RCC subtyping depends on the cell of origin, morphology, growth pattern, and histochemical and molecular characteristics [4-7]. The subtypes include clear cell RCC (which accounts for 75% to 80% of cases) [1,2]. PRCC is the most common non-clear cell RCC [1].

PRCC originates from the proximal tubule and accounts for approximately 10% to 15% of all RCC cases. It has unique histopathology, molecular alterations, and clinical presentation that might influence treatment response to systemic agents. PRCC is classified as type I or type II based on histopathologic features [1,2].Type I PRCC presents with stage I or II disease and has a favorable prognosis. Most cases are sporadic, and 10% to 20% of cases are associated with dysregulation of the mesenchymal-epithelial transition (MET) pathway due to somatic mutation. The hepatocyte growth factor receptor MET is a membrane-associated receptor tyrosine kinase implicated in developing many malignancies, including PRCC. Uncontrolled MET signaling can occur through several molecular mechanisms, including mutations. Activating mutations in the kinase domain of MET are found in the majority of hereditary PRCC cases and approximately 5% to 13% of sporadic PRCC cases [8].

Type II PRCC is associated with an aggressive course and advanced stage at presentation and has a less favorable prognosis than type I. It has been linked to hereditary leiomyomatosis and renal cell cancer syndrome, caused by a mutation in the gene for fumarate hydrates [3,9]. The currently available therapeutic options for PRCC, regrettably, provide only limited clinical benefits, depending on the extent of the disease. Surgery is the treatment of choice for localized disease (stage I to III), while locally advanced or metastatic disease often necessitates systemic treatment.

Evolving either checkpoint inhibitor immunotherapy or a VEGF receptor inhibitor as initial therapy may significantly improve survival [10]. Clear-cell RCC is typically more responsive to TKIs and ICIs. However, PRCC often exhibits resistance to available treatments [11,12]. Sunitinib is an effective oral initial therapy that improves progression-free survival (PFS) and objective response rates (ORR) in patients with PRCC in phase II clinical trials [13,14]. Sunitinib works by inhibiting several receptor tyrosine kinases involved in cancer growth, metastasis, and neoangiogenesis, including platelet-derived growth factor, VEGF, and other receptors. Sunitinib could be given on an individualized schedule based on tolerance [11]. Cabozantinib, another promising TKI with the advantage of targeting the MET receptor, showed prolonged PFS and ORR compared to sunitinib in a phase II trial [15].

The effectiveness of first-line immunotherapy-based treatments in non-clear cell RCC has been demonstrated in multiple phase II and observational retrospective studies [16-18]. A phase III randomized trial is currently ongoing, looking at first-line nivolumab and ipilimumab versus sunitinib in non-clear cell RCC. More extensive trials are needed to identify additional molecular biomarkers predicting treatment responses.

## Conclusions

Sunitinib remains an excellent option in a subset of patients with metastatic type II PRCC. This case demonstrated prolonged responses to sunitinib lasting for three years, including an initial CR. To the best of our knowledge, this is the first case of PRCC with CR to sunitinib. Further studies are still needed to validate this conclusion and provide more references for detailed strategies for individualized treatment.

## References

[1] (2022). Papillary renal cell carcinoma. https://www.cancer.gov/pediatric-adult-rare-tumor/rare-tumors/rare-kidney-tumors/papillary-renal-cell-carcinoma.

[2] Deng J, Li L, Xia H (2019). A comparison of the prognosis of papillary and clear cell renal cell carcinoma: Evidence from a meta-analysis. Medicine (Baltimore).

[3] Delahunt B, Eble JN (1997). Papillary renal cell carcinoma: a clinicopathologic and immunohistochemical study of 105 tumors. Mod Pathol.

[4] Bukhari N, Al-Badr S, AlNaimi M, Azam F (2020). Sunitinib: an unusual cause of pneumothorax in a patient with metastatic chromophobe renal cell carcinoma. Cureus.

[5] Siegel RL, Miller KD, Fuchs HE, Jemal A (2021). Cancer statistics, 2021. CA Cancer J Clin.

[6] (2021). Cancer of the kidney and renal pelvis - cancer stat facts. https://seer.cancer.gov/statfacts/html/kidrp.html.

[7] van de Pol JA, George L, van den Brandt PA, Baldewijns MM, Schouten LJ (2021). Etiologic heterogeneity of clear-cell and papillary renal cell carcinoma in the Netherlands Cohort Study. Int J Cancer.

[8] Marcon J, Graser A, Horst D (2020). Papillary vs clear cell renal cell carcinoma. Differentiation and grading by iodine concentration using DECT-correlation with microvascular density. Eur Radiol.

[9] Ciccarese C, Iacovelli R, Brunelli M (2017). Addressing the best treatment for non-clear cell renal cell carcinoma: a meta-analysis of randomised clinical trials comparing VEGFR-TKis versus mTORi-targeted therapies. Eur J Cancer.

[10] Ravaud A, Oudard S, De Fromont M (2015). First-line treatment with sunitinib for type 1 and type 2 locally advanced or metastatic papillary renal cell carcinoma: a phase II study (SUPAP) by the French Genitourinary Group (GETUG)†. Ann Oncol.

[11] Armstrong AJ, Halabi S, Eisen T (2016). Everolimus versus sunitinib for patients with metastatic non-clear cell renal cell carcinoma (ASPEN): a multicentre, open-label, randomised phase 2 trial. Lancet Oncol.

[12] Deeks ED, Raymond E (2011). Sunitinib: in advanced, well differentiated pancreatic neuroendocrine tumors. BioDrugs.

[13] Oudard S, Geoffrois L, Guillot A (2016). Clinical activity of sunitinib rechallenge in metastatic renal cell carcinoma-Results of the REchallenge with SUnitinib in MEtastatic RCC (RESUME) Study. Eur J Cancer.

[14] Bukhari N, Winquist E (2017). Case: secondary polycythemia due to pazopanib in patients with metastatic renal cell carcinoma. Can Urol Assoc J.

[15] Tannir NM, Jonasch E, Albiges L (2016). Everolimus versus sunitinib prospective evaluation in metastatic non-clear cell renal cell carcinoma (ESPN): a randomized multicenter phase 2 trial. Eur Urol.

[16] McDermott DF, Lee JL, Ziobro M (2021). Open-label, single-arm, phase ii study of pembrolizumab monotherapy as first-line therapy in patients with advanced non-clear cell renal cell carcinoma. J Clin Oncol.

[17] Koshkin VS, Barata PC, Zhang T (2018). Clinical activity of nivolumab in patients with non-clear cell renal cell carcinoma. J Immunother Cancer.

[18] Tykodi SS, Gordan LN, Alter RS (2022). Safety and efficacy of nivolumab plus ipilimumab in patients with advanced non-clear cell renal cell carcinoma: results from the phase 3b/4 CheckMate 920 trial. J Immunother Cancer.

